# Full-field hard X-ray nano-tomography at SSRF

**DOI:** 10.1107/S1600577523003168

**Published:** 2023-05-05

**Authors:** Fen Tao, Jun Wang, Guohao Du, Bo Su, Ling Zhang, Chen Hou, Biao Deng, Tiqiao Xiao

**Affiliations:** a Shanghai Synchrotron Radiation Facility, Shanghai Advanced Research Institute, Chinese Academy of Sciences, Shanghai 201204, People’s Republic of China; b Shanghai Institute of Applied Physics, China Academy of Science, Shanghai 201800, People’s Republic of China; c University of Chinese Academy of Science, Beijing 100084, People’s Republic of China; University of Tokyo, Japan

**Keywords:** X-ray nano-imaging, ellipsoidal capillary, synchrotron radiation facility, spatial resolution

## Abstract

An in-house designed transmission X-ray microscopy (TXM) instrument has been developed and commissioned at beamline BL18B of the Shanghai Synchrotron Radiation Facility (SSRF). BL18B is a hard (5–14 keV) X-ray bending-magnet beamline recently built with sub-20 nm spatial resolution in TXM. There are two kinds of resolution mode: one based on using a high-resolution-based scintillator-lens-coupled camera, and the other on using a medium-resolution-based X-ray sCMOS camera. Here, a demonstration of full-field hard X-ray nano-tomography for high-*Z* material samples (*e.g.* Au particles, battery particles) and low-*Z* material samples (*e.g.* SiO_2_ powders) is presented for both resolution modes. Sub-50 nm to 100 nm resolution in three dimensions (3D) has been achieved. These results represent the ability of 3D non-destructive characterization with nano-scale spatial resolution for scientific applications in many research fields.

## Abstract 2

A demonstration of full-field hard X-ray nano-tomography for high- and low-*Z* material samples is presented for the two TXM resolution modes developed at beamline BL18B at SSRF.

## 1. Introduction

Full-field transmission X-ray microscopy (TXM) is a powerful technology for non-destructive three-dimensional (3D) imaging at the nanometre scale (Wang *et al.*, 2012 ▸; Chao *et al.*, 2005 ▸). Owing to the short wavelength of hard X-rays and their ability to penetrate matter, compared with soft X-ray TXM, hard X-ray TXM provides larger penetration and depth of focus (Yuan *et al.*, 2012 ▸). Nano-tomography is an important application of the TXM system coupled with a rotational sample stage such that 3D internal structural information at the nanometre scale can be generated. Compared with typical micro-scale computed tomography (µCT) systems, synchrotron-based hard X-ray nano-tomography with a higher spatial resolution (Kalbfleisch *et al.*, 2022 ▸; Suzuki *et al.*, 2016 ▸; Weitkamp *et al.*, 2017 ▸) has a lot of applications in many fields, including energy materials (Lim *et al.*, 2017 ▸; Zhang *et al.*, 2022 ▸; Li *et al.*, 2022 ▸), biomedical research (Suuronen *et al.*, 2022 ▸) and other areas (Larsson *et al.*, 2019 ▸).

We have built an in-house-designed hard X-ray full-field nano-tomography instrument at BL18B of the Shanghai Synchrotron Radiation Facility (SSRF) (Tao *et al.*, 2022 ▸). Beamline BL18B has two kinds of resolution mode: high-resolution mode based on a scintillator-lens-coupled camera, and medium-resolution mode based on an X-ray sCMOS camera. High-resolution mode specializes in spatial resolution and is able to identify resolutions of 20 nm or better, and medium-resolution mode specializes in both spatial resolution and exposure time in a high level of balance. The TXM endstation of BL18B has been commissioned and the microscope has the capability of generating 2D images with sub-20 nm and sub-70 nm resolution for the high- and medium-resolution modes, respectively. An imaging unit can be selected in accordance with the desired resolution. To evaluate the 3D resolution and image quality of nano-tomography with both resolution modes, we conducted nano-tomography experiments on three different types of samples. In this paper, we present a demonstration of full-field hard X-ray nano-tomography for high-*Z* material samples (*e.g.* Au powder, battery particles) and low-*Z* material samples (*e.g.* SiO_2_ particles). These results represent the ability of 3D non-destructive characterization with nano-scale spatial resolution for scientific applications in many research fields. In particular, for medium-resolution mode, high-quality imaging of low-*Z* materials can be achieved at low energy (5–6 keV).

## 2. Beamline and endstation overview

**Figure 1:**
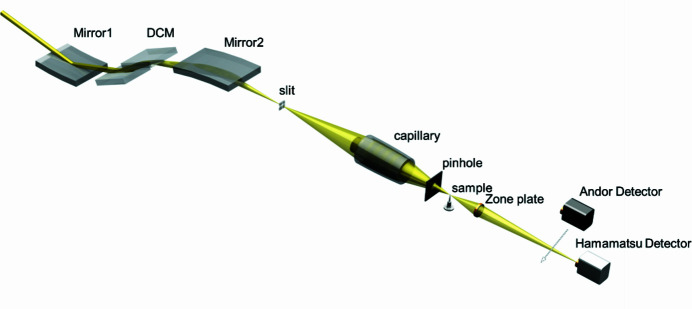
General schematic of the layout of beamline BL18B at SSRF.

Beamline BL18B is a hard X-ray nano-tomography beamline located at sector 18B of SSRF. The source is a bending magnet (BM) (3.5 GeV, 1.27 T, 300 mA). The primary layout of TXM optics at BL18B is shown in Fig. 1 ▸. Along the incident X-ray path, beamline BL18B includes a parabolic cylinder mirror (M1), double-crystal monochromator (DCM) with a set of standard Si(111) crystals, a toroidal mirror (M2) and slit. The synchrotron X-ray beam from the BM source is collimated vertically by M1, followed by a Si(111) double-crystal monochromator, and then focused by M2. The acceptance angle of the M1 mirror is 0.4 mrad (horizontal) × 0.22 mrad (vertical). The grazing-incident angle for the M1 mirror is 4.4 mrad. The slit is set at the focus point of M2. The monochromator selects the X-ray energy required in the photon energy range 5–14 keV. The TXM operates with the sample principle as a visible light microscope, consisting of a source providing the radiation (BM source and beamline), a condenser (ellipsoidal mono-capillary) relaying the radiation onto an object (sample), an X-ray objective lens (Fresnel zone plate) and X-ray imaging detectors.

There are two kinds of X-ray imaging detector used in the beamline: (i) a Hamamatsu C12849-111U camera, forced-air cooled with a 1:1 fiber-optic channel plate and an sCMOS chip of 6.5 µm × 6.5 µm pixel size [2048 (H) × 2048 (V) pixels]; and (ii) an X-ray scintillator-lens-coupled camera. The X-ray image is converted to the visible range by the thin scintillation crystal (100 µm-thick LUAG), re-magnified by the 2×, 4× and 10× optical objective and then measured by the water-cooled sCMOS 2048 × 2048 pixel detector (Andor, ZYLA 4.2 Plus).

**Figure 2:**
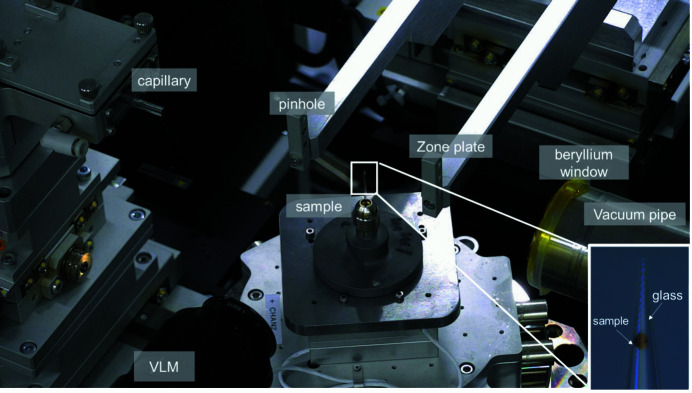
Photograph of the TXM endstation at SSRF BL18B, with the mounting of the sample shown in the bottom-right corner.

Fig. 2 ▸ shows a photograph of the TXM endstation at BL18B of SSRF. The path of the X-rays through the endstation can be summarized as follows. Illumination shaped by a condenser is focused onto a ∼15 µm spot on the sample. A 200 µm pinhole is placed 50 mm upstream of the sample to reduce the background signal. An X-ray projection is generated by the sample placed 93 mm downstream from the condenser, which is magnified by a zone plate objective, and then onto to the X-ray imaging detector system. An ellipsoidal mono-capillary-based in-house design is used as the condenser to focus the beam onto the sample (Tao *et al.*, 2017 ▸). The condenser illuminates a spot on the sample providing a field of view (FOV) of approximately 20 µm in diameter at 8 keV with a central stop that provides a hollow cone illumination. The rotation stage used for nano-tomography is an air-bearing rotation stage with very small run-out error and the sample is centered using an XY assembly of two positioners. Two sets of Fresnel zone plates (FZPs), with an outermost width of 25 nm (focal length *f*_FZP_ = 20.2 mm @ 8 keV) and 40 nm (*f*_FZP_ = 25.8 mm @ 8 keV) are used as the objective lens for high-resolution mode (the magnification factor can vary from about 500× to 1030×) and medium-resolution mode (the magnification factor can vary from about 145× to 185×). In order to reduce the attenuation of air along the X-ray path, a vacuum pipe is installed between the zone plate and detector, where a 1 × 10^−1^ Pa low vacuum is maintained. A visible-light microscope (VLM) consisting of a 12× optical microscope objective is mounted on an XYZ stage for pre-alignment of the sample. This VLM allows positioning of a particular region of interest of the sample in the X-ray field of view, which is small compared with the sample size. In particular, the sample is mounted on top of a glass tube of size smaller than the sample diameter so that a single particle can be stuck. Then, imaging of the individual sample can be realized. Mounting of the sample is shown in the bottom-right corner of Fig. 2 ▸.

## 3. TXM spatial resolution

There are two kinds of resolution mode at SSRF BL18B: high-resolution mode based on a scintillator-lens-coupled Andor ZYLA 4.2 Plus camera, and medium-resolution mode based on a Hamamatsu C12849-111U camera. The high-resolution detector can travel from 1300 to 2100 mm downstream of the zone plate and the demagnified pixel size is about 6–13 nm. High-resolution mode specializes in reaching the limit of spatial resolution for the TXM system and an ultra-limited spatial resolution can be achieved sub-20 nm. At high resolution, the exposure time for each 2D projection image is set from 3 to 10 s, and therefore the total acquisition time for a 3D tomography dataset can reach at least 20–30 min. Reducing the data acquisition time is highly desirable because it improves the temporal resolution for measurements that focus on the sample dynamic transition (Ge *et al.*, 2018 ▸). The lower total dose of the X-ray irradiation with short X-ray exposure time under the same conditions reduces the effect on the irradiation-sensitive sample. Thus, we develop a medium-resolution mode with sub-70 nm spatial resolution using a Hamamatsu C12849-111U camera placed at 3700 mm downstream of the zone plate for which the demagnified pixel size is ∼30 nm. Compared with high-resolution mode, the ultimate exposure time is decreasing to 0.3 s @ 8 keV for the 2D projection images and the total acquisition time for the 3D reaches about 1–2 min in medium-resolution mode (more than ten times faster than in high-resolution mode). Considering the attenuation of air and low efficiency of the detector at low energy, imaging is impossible for high-resolution mode. However, the exposure time of 5 s @ 5.2 keV is sufficient for low-*Z* material in medium-resolution mode. For specific experiments, the desirable modes can be selected depending on the 2D projection imaging requirement. In both systems, the low-vacuum pipe, water-cooled detector and fiber-optic channel plate detector are utilized to improve the signal-to-noise ratio of the image.

### 3.1. Standard Siemens star pattern

**Figure 3:**
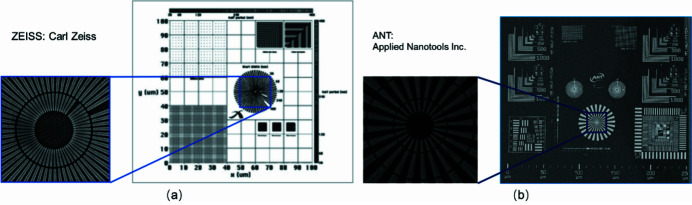
Design drawing of the two resolution targets and the corresponding SEM images of the Siemens star pattern. (*a*) 30 nm finest features from ZEISS; (*b* 50 nm finest features from ANT.

Imaging systems require precise measurement of their spatial resolution, image distortion and magnification calibration. Knowledge of these parameters is essential for specifying the quality of a certain optical system. To accommodate the varying resolution and penetration of X-ray imaging systems, the structure width and absorption of a known reference pattern have to be matched to the system parameters. Here, two high-resolution calibration standard test patterns (Siemens stars) are applied to evaluate the spatial resolution and the performance for both resolution modes in TXM. The isotropic test pattern of the Siemens star offers help for resolution tests, modulation transfer measurements, length scale calibration and field distortion measurements. For the two resolution modes, two Siemens Au star test patterns with different finest features size were used for the spatial resolution tests. One standard Siemens star pattern (ZEISS, Carl Zeiss) with smallest feature size of 30 nm (the width of an innermost radial line, 30 nm half-pitch) and ∼200 nm-thick gold fabricated on 110 nm silicon nitride membrane is used for testing the ultra-spatial resolution in high-resolution mode. Another standard Siemens star pattern (ANT, Applied Nanotools Inc.) with smallest feature size of 50 nm (the width of an innermost radial line, 50 nm half-pitch) and ∼600 nm-thick gold fabricated on 100 nm silicon nitride membrane was used for testing the spatial resolution in medium-resolution mode. The scanning electron microscopy (SEM) images of the Siemens star patterns are shown in Fig. 3 ▸. The 30 nm and 50 nm finest features can be easily distinguished for the ZEISS and ANT test patterns, respectively.

### 3.2. TXM spatial resolution test

TXM spatial resolution was tested both in high and medium mode. Similar to the objective lens for optical systems, two kinds of FZPs were switched in the TXM system according to the target resolution modes. A gold FZP with an outermost zone width of Δ*r* = 25 nm and diameter of 125 µm (with a thickness of 600 nm) was used in the TXM system for testing the ultimate resolution in high-resolution mode. The X-ray energy was set as 8 keV for high mode and the absorption for the ∼200 nm-thick gold Siemens star pattern was 8%. The other gold FZP with outmost zone width of Δ*r* = 40 nm, diameter of 125 µm and total thickness of 600 nm (including the Au layer on silicon nitride membrane) was switched for medium-resolution calibration. An X-ray energy of 5.2 keV was used for medium mode and the absorption for the ∼600 nm-thick gold Siemens star pattern was 52%.

**Figure 4:**
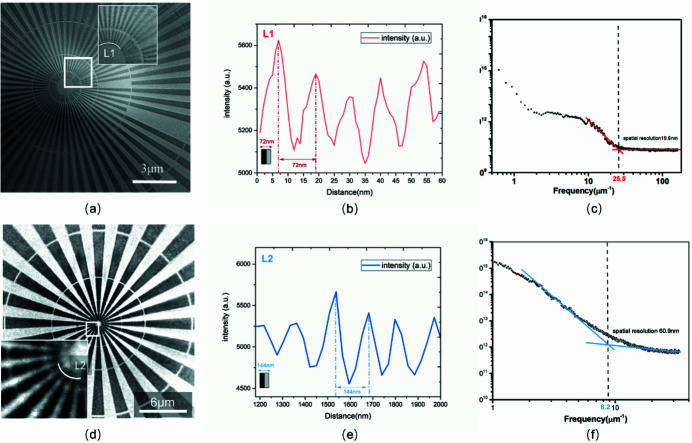
Results of the Siemens star pattern tests for the different resolution modes. (*a*–*c*) Results for high-resolution mode. (*a*) Siemens star pattern with 30 nm finest feature size as indicated by circle L1 by absorption contrast, with an insert of an enlarged quarter of the center in high-resolution mode at 8 keV. (*b*) Line profile of L1. (*c*) Power spectrum density analysis on the star patterns by absorption contrast (*a*). (*d*–*f*) Results for medium-resolution mode. (*d*) Siemens star pattern with 50 nm finest feature size as indicated by circle L2 by absorption contrast, with an insert of an enlarged quarter of the center in medium-resolution mode at 5.2 keV. (*e*) Line profile of L2. (*f*) Power spectrum density analysis on the star patterns by absorption contrast (*d*).

Fig. 4 ▸(*a*) shows an absorption contrast image taken at 8 keV of the Siemens star pattern with exposure time of 30 s, with an insert of an enlarged quarter of the center. The inner circle (L1) of the Siemens star pattern has the smallest line width of ∼36 nm (∼72 nm period). In Fig. 4 ▸(*b*), the line profile along L1 is generated by interpolation of the nearest adjacent pixel values, indicating the contrast of the image. A more accurate analysis of the whole area of Fig. 4 ▸(*a*) is conducted by the accepted technique (Chao *et al.*, 2005 ▸) of radial power spectrum density (RPSD) (Chen *et al.*, 2011 ▸). Fig. 4 ▸(*c*) shows that the power spectrum of the sample cuts off at a spatial frequency of 25.5 µm^−1^, corresponding to a full period of about 39.2 nm. Thus, the TXM spatial resolution of absorption in high-resolution mode was 19.6 nm. Similar results for the medium-resolution mode are shown in Figs. 4 ▸(*d*)–4(*f*): the image of the Siemens star pattern was taken at 5.2 keV with an exposure time of 10 s, and the inner circle (L2) of the Siemens star pattern has a line width of ∼72 nm (∼144 nm period), with an insert of an enlarged quarter of the center also shown. The power spectrum of the sample cuts off at a spatial frequency of 8.2 µm^−1^, corresponding to a period of about 121.8 nm. Therefore the spatial resolution of absorption in medium-resolution mode was recognized as 60.9 nm. From the overall image of the FOV, as shown in Figs. 4 ▸(*a*) and 4(*d*), the width of the FOV of the high and medium mode were 13 µm and 20 µm, respectively. The FOV is smaller in the high-resolution mode than in the middle one.

The spatial resolution of TXM for two kinds of resolution modes was investigated with the results shown above. The 30 nm finest feature in the center of the star bar is clearly distinguished in high-resolution mode, and the limited spatial resolution can be sub-20 nm. For the medium resolution mode, a sub-70 nm spatial resolution could be used. At the same time, the Siemens star pattern images were acquired by both TXM [Figs. 4 ▸(*a*)–4(*d*)] and SEM [Figs. 3 ▸(*a*)–3(*b*)]. The shape and feature size shown in the results are consistent with each other, which illustrates the reliability of the TXM system. The experimental results show that the TXM system can achieve a spatial resolution from 30 to 100 nm.

## 4. Full-field X-ray nano-tomography

### 4.1. Referenced sample nano-tomography experiment

To evaluate the absorption-contrast imaging capacities of the TXM system for the two resolution modes, certain reference samples were chosen for relevant 3D nano-tomography. Gold particles (high-*Z* material, density 19.37 g cm^−3^) were imaged in high-resolution mode to evaluate the spatial resolution quality. Then SiO_2_ powders (low-*Z* material, density 2.3 g cm^−3^) were imaged in medium-resolution mode to test the image reliability. Finally, the 3D reconstruction results were compared with the corresponding SEM observations.

**Figure 5:**
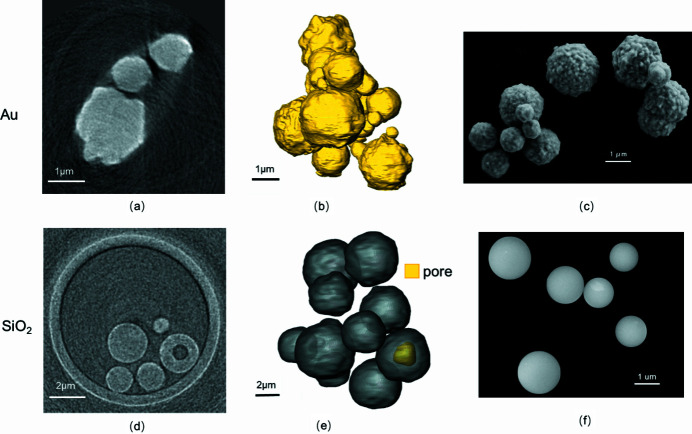
High- and low-*Z* reference sample nano-imaging by the two resolution modes. (*a*–*c*) Image of a high-*Z* material – gold particles. (*a*) A 2D slice of the gold particles with 20 µm field of view taken at 8 keV. (*b*) A rending of the 3D structure of a local region. (*c*) The same batch of gold particles imaged with SEM. (*d*–*e*) Image of a low-*Z* material – SiO_2_ powder. (*d*) A 2D slice of the SiO_2_ with 20 µm field of view taken at 5.2 keV. (*e*) A rending of the 3D structure of a local region. (*f*) The same batch of SiO_2_ powders imaged with SEM.

Several gold particles were imaged with 8 keV X-rays in high-resolution mode with demagnified pixel size of 10 nm. According to the theoretical estimation, the absorption for 1–3 µm-thick gold is from 33 to 70%. Such absorption contrast is preferential for precise observation. A total of 180 projection images were taken from 0 to 180° in high-resolution mode at 1° step^−1^. The exposure time for each 2D projection image was 5 s and the total time for acquiring the projection stack was 20 min. The field of view was about 20 µm × 20 µm, and a typical reconstructed slice for the gold particles is presented in Fig. 5 ▸(*a*). Fig. 5 ▸(*b*) shows gold particles of different sizes reconstructed using the *Avizo Fire* software (Visualization Sciences Group, Bordeaux, France). The size of the particles ranged from 0.5 to 3 µm and the particles have rough surface with a height fluctuation of around 0.3–1 µm. All of these reconstruction results are consistent with those observed via SEM.

SiO_2_ powders were imaged with 5.2 keV X-rays in medium-resolution mode with demagnified pixel size of 29 nm. Under such an X-ray energy, the absorption for a 2 µm-diameter SiO_2_ particle is estimated to be 13%, which is slightly lower for absorption-contrast imaging. A total of 180 equiangular projections between 0 and 180° were obtained with an exposure time of 5 s. A slice of several SiO_2_ powders is shown in Fig. 5 ▸(*d*) and the wall of the glass tube was also able to be distinguished clearly. Such results show the reliability of the medium-resolution mode in low-*Z* materials. More importantly, there is no need to increase the exposure time to enhance the image contrast or adopt any special contrast-improvement algorithm. Also, a 0.8 µm cavity was observed inside a SiO_2_ powder which predicted the potential of 3D non-destructive characterization with nano-scale spatial resolution.

### 4.2. Initial application in lithium-ion battery

**Figure 6:**
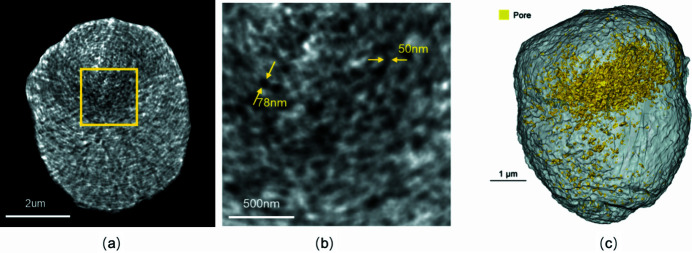
Image of an NCM622 battery in high-resolution mode at 8 keV. (*a*) A 2D slice of a 10 µm-diameter particle of the charged NCM622 material. (*b*) Enlarged view of the yellow square in (*a*). (*c*) 3D reconstruction of NCM622.

Li-ion batteries (LIBs) are electrochemical systems and there always exists pursuit for high energy density and good cycling performance to fulfill demands in industrial applications. Chemomechanical breakdown is an important fading mechanism in LIBs that affects the properties. Thus, quantitative analysis of crack formation becomes the key method for battery research and TXM gradually plays an important role in the nano-scale characterization of battery components (Alemu & Wang, 2018 ▸; Wei *et al.*, 2020 ▸; Jiang *et al.*, 2020 ▸). The cathode of a charged LiNi_0.6_Co_0.2_Mn_0.2_O_2_ (NCM622) battery was separated from the current collector and a 6 µm particle was imaged with 8 keV X-rays in high-resolution mode. The *Avizo Fire* software was used for segmentation of the isolated pores according to the algorithm. A total of 180 projection images were taken from 0 to 180° and the exposure time for each 2D projection image was 5 s. Figs. 6 ▸(*a*)–6(*b*) show 2D cross sections of the reconstructed image, and the nano-scale crack can be easily discerned and segmented by gray-scale threshold difference. The local size and shape of the cracks can be measured according to the pixel sizes. The yellow arrows show the limited length of the pore in Fig. 6 ▸(*b*). With the help of the *Avizo* software, the 3D configuration of the particle as well as the inner crack network can be reconstructed, as shown in Fig. 6 ▸(*c*). The crack initiated from the center of the particle and branched outwards. The volumes of the crack and the battery particle are measured as 1.9 µm^3^ and 75.4 µm^3^, respectively, corresponding to a porosity of 2.5%. This work highlights the potential of TXM at BL18B in nano-scale characterization, especially for nondestructive visualization of materials.

## 5. Conclusions

Two resolution modes for TXM have been developed at the BL18B beamline at SSRF. High-resolution mode and medium-resolution mode are based on two kinds of detector and FZP. According to the Siemens star pattern tests, the two modes can achieve sub-20 nm and sub-70 nm spatial resolution, respectively. High-*Z* material samples (gold particles) and low-*Z* material samples (SiO_2_ powders) have been tested for both modes, and 3D reconstructions of the samples have been achieved and compared with results observed via SEM. The TXM system has demonstrated its ability to handle ultimate high-resolution imaging and reliable medium-resolution experiments for low-*Z* materials at low energy (5–6 keV). Finally, nano-scale crack configurations in battery material are presented and both morphology and size features of cracks can be analyzed quantitatively. The instrument is now entering user operation and will be put to use as an effective tool in the field of nano-scale research. In addition, X-ray absorption near-edge structure (XANES) and TXM-XANES techniques are part of our ongoing work which have the unique capability of producing chemical composition images of battery materials.
